# Synthesis of Straw-Based Hydrothermal Carbonation Carbon and Its Photocatalytic Removal of Cr(VI) and Microcystin-LR

**DOI:** 10.3390/molecules30224399

**Published:** 2025-11-14

**Authors:** Yu Luo, Xunxian Chen, Zhen Wan, Yingming Chen

**Affiliations:** 1 Key Laboratory of Catalysis and Materials Science of the State Ethnic Affairs Commission and Ministry of Education, College of Resources and Environmental Science, South-Central Minzu University, Wuhan 430074, China; cy921127@outlook.com; 2 Foshan Water Industry Group Co., Ltd., Foshan 528000, China; 13827722820@sohu.com

**Keywords:** straw-based HTCC, acid modification, photocatalyst, Cr(VI) reduction, microcystin-LR

## Abstract

As a cost-effective and environmentally benign photocatalyst, hydrothermal carbonation carbon (HTCC) has been extensively studied in the fields of resource utilization and environmental remediation. In this study, HTCC photocatalysts with efficient photocatalytic performances were prepared from straw using acid modification under hydrothermal conditions. The as-prepared HTCC photocatalysts were applied to the degradation of microcystin-LR and the reduction of aqueous Cr(VI). The critical role of acid modification in the photocatalytic performances of the HTCC photocatalysts was systematically investigated. The results demonstrated that acid-modified photocatalysts exhibited a significantly enhanced removal efficiency for Cr(VI) and microcystin-LR under visible light irradiation. A series of characterization techniques, including Raman spectroscopy and N_2_ adsorption–desorption analysis, revealed that the superior photocatalytic activities of acid-modified HTCC could be attributed to its higher aromatization level, enhanced light-harvesting ability, and increased concentration of active sites compared with pristine HTCC. Furthermore, electron spin resonance (ESR) and trapping experiments indicated that hydrogen radicals (·H) served as the primary active species in the photocatalytic Cr(VI) reduction of straw-based HTCC. This work provides both practical and theoretical insights into the resource utilization of agricultural waste and the remediation of environmental pollution.

## 1. Introduction

Malodorous black water usually contains two prominent categories of hazardous contaminants—microcystins and heavy metals [1,2,3]. Hexavalent chromium (Cr(VI)), a Group 1 IARC carcinogen, is especially notorious for its high mobility, persistence, and toxicity. The extensive use of chromium (Cr) in modern industries such as electroplating, metallurgy, and textiles has led to the widespread elimination of hazardous Cr(VI), thereby giving rise to a serious threat to aquatic ecology and public health [4,5,6,7,8]. Additionally, as potent cyclic hepatotoxins, microcystins generated by cyanobacterial proliferation have become a global concern due to their serious risks to human health [9,10,11,12]. Among various microcystins, microcystin-LR is one of the most toxic and prevalent variants worldwide due to its ability to damage to miRNA expression regulation and inhibit the activity of protein phosphatases in humans [13,14,15,16,17]. Therefore, it is of great importance that feasible technology should be adopted to remove both Cr(VI) and microcystin-LR.

Recently, photocatalysis has garnered significant attention for the remediation of pollutants in malodorous black water, such as heavy metals and microcystins, and has demonstrated its promising application potential [18,19,20,21,22]. The development of efficient and eco-friendly novel photocatalysts has been the primary objective of research. In particular, HTCC photocatalysts, a group of functional carbon materials mainly derived from biomass and formed by mimicking natural coalification processes, not only facilitate the removal of pollutants like Cr(VI) and microcystin-LR but also enable the resourceful utilization of vast agricultural and forestry waste streams, thereby contributing to the realization of a green and low-carbon economy [23,24,25,26]. HTCC photocatalysts have exhibited advantages such as a uniform particle size, stable physicochemical properties, and abundant surface oxygen-containing functional groups. However, HTCC photocatalysts prepared by conventional methods have usually demonstrated poor photocatalytic activities, primarily due to the scarcity of active sites on the catalysts’ surfaces, leading to the high recombination efficiency of photogenerated electron–hole pairs [27,28,29]. To address this issue, many modification strategies for HTCC have been developed such as ionic doping, metal deposition, heterostructure fabrication, and acid modification [30,31,32,33]. For example, Hu et al. reported that iodine dopants facilitated charge transfer, thus increasing the conductivity and activity of HTCC [34]. Wang et al. fabricated CoFe_2_O_4_/HTCC composites which exhibited superior photocatalytic inactivation toward Escherichia coli K-12 under visible light irradiation [35]. Among these strategies, acid modification has attracted significant interest owing to its remarkable ability to boost the catalyst’s efficiency in producing reactive species. For instance, Xu et al. found that acid etching improved the charge transfer rate and facilitated the generation of superoxide radicals, which in turn led to a superior photocatalytic performance [29]. In a separate study, Xu et al. prepared a modified HTCC photocatalyst from cellulose via a sulfuric acid-assisted hydrothermal method. The acid-modified HTCC photocatalyst demonstrated a superior ability to activate molecular oxygen for generating superoxide radicals, which contributed to its enhanced photocatalytic performance in the reduction of Cr(VI) compared with its unmodified counterpart. [36].

Herein, in this work, acid-modified HTCC photocatalysts were fabricated from straw precursors using a one-step hydrothermal process, with the aim of regulating its properties through the addition of different concentrations of nitric acid. On the basis of various characterizations such as UV–vis DRS, Raman, and ESR, we demonstrated that the acid-modified HTCC photocatalyst exhibited a higher aromatization level, improved light-harvesting ability, and increased concentration of active sites compared with its unmodified counterpart. As a result, the photocatalytic removal rate of Cr(VI) and microcystin-LR over the acid-modified HTCC photocatalysts were dramatically enhanced, both being about six-fold times higher than that of pristine HTCC. Moreover, ESR and probe experiments confirmed that the ·H radicals are a key active species generated during the photocatalytic Cr(VI) reduction of the as-prepared HTCC photocatalysts. Our finding provides valuable insights into the application of biomass-derived photocatalysts in environmental remediation.

## 2. Results and Discussion

### 2.1. Structure and Morphology

**Figure 1:**
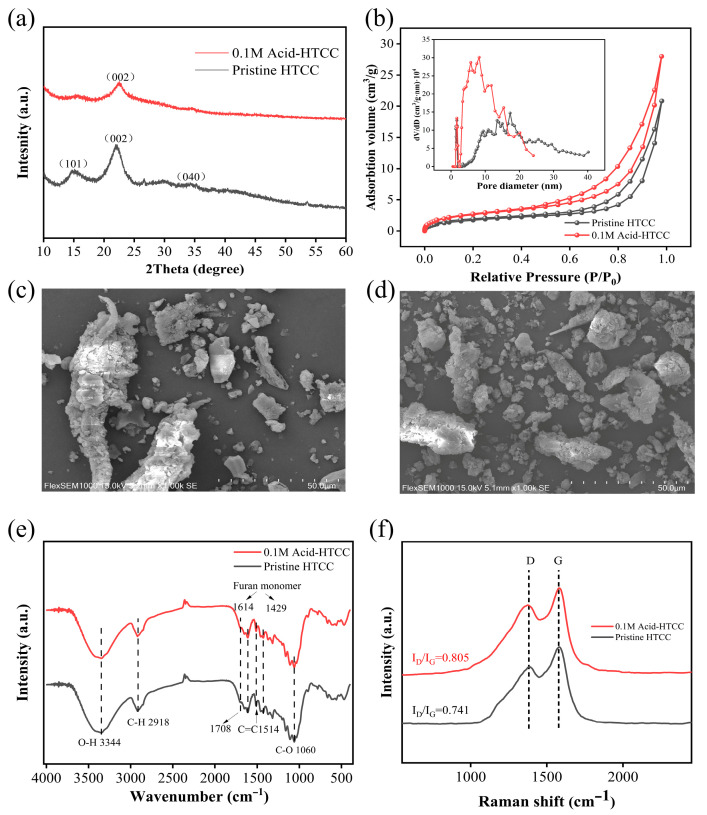
(**a**) XRD patterns and (**b**) N_2_ adsorption and desorption curves of the pristine HTCC and 0.1 M Acid-HTCC. The pore size distributions of the samples are given in the inset of (**b**). SEM images of the (**c**) pristine HTCC and (**d**) 0.1 M Acid-HTCC. (**e**) FT-IR and (**f**) Raman spectra of the pristine HTCC and 0.1 M Acid-HTCC.

Figure 1a shows the XRD patterns of the 0.1 M Acid-HTCC and pristine HTCC. The peaks observed at 2θ of 14.9°, 22.1°, and 33.9° in the pattern of the pristine HTCC could be attributed to the (101), (002), and (040) planes of the carbon fiber structure [37]. The characteristic peaks at 26.6° and 29.7° were assigned to the cellulose from the precursor of straw (PDF#03-0226), which indicates the incomplete carbonization of the straw during the hydrothermal reaction and the preservation of partial cellulose structures. In contrast, the crystallinity of the acid-modified photocatalyst (0.1 M Acid-HTCC) decreased significantly, primarily due to the addition of nitric acid promoting cellulose hydrolysis and the polymerization of resulting hydrolyzates during the hydrothermal process, thereby forming more amorphous aromatic carbon.

Figure 1b shows the N_2_ absorption–desorption isotherms and corresponding pore size distributions of 0.1 M Acid-HTCC and pristine HTCC. From the figure, both of the two samples exhibited Type IV isotherms and H3 hysteresis loops, confirming their layered mesoporous structures [38]. The specific surface areas, determined by the Brunauer–Emmett–Teller (BET) methods, were 6.6 m^2^ g^−1^ for pristine HTCC and 9.9 m^2^ g^−1^ for 0.1 M Acid-HTCC (Table 1). The larger surface area of the acid-modified photocatalyst suggests that the acid treatment partially collapsed the cellulose framework, which is consistent with the above XRD results. The SEM results further confirmed this observation. As shown in Figure 1c,d, both the pristine HTCC and 0.1 M Acid-HTCC exhibited an irregular and blocky morphology. However, the acid-modified sample (0.1 M Acid-HTCC) showed a significantly increased population of smaller particles (≤ 5 μm) in contrast to the pristine HTCC. As revealed by the SEM images recorded at higher magnification in Figure S1, these blocky particles in both the pristine HTCC and 0.1 M Acid-HTCC consisted of a straw-derived fibrous skeleton with amorphous carbon particles embedded on the surface. TEM analysis also corroborated these morphological characteristics. As shown in Figure S2, the surfaces of blocky particles from 0.1 M Acid-HTCC were covered with amorphous carbon nanoparticles smaller than 50 nm, which prevented the observation of lattice fringes associated with the fibrous structure in HRTEM. Furthermore, the pore size distributions derived from the Barrett–Joyner–Halenda (BJH) desorption curves revealed that the acid-modified photocatalyst of 0.1 M Acid-HTCC possessed smaller pores, with a maximum centered at 8.28 nm, compared with the pristine HTCC, which can be ascribed to the higher content of amorphous carbon nanoparticles on the 0.1 M Acid-HTCC surface.

The FT-IR spectra of the pristine HTCC and 0.1 M Acid-HTCC are shown in Figure 1e. From the figure, both of the two samples exhibited similar features, with characteristic absorptions identified as follows: O-H and C-H stretching vibrations at 3344 and 2918 cm^−1^ respectively; C=O stretching at 1708 cm^−1^; furan monomer vibrations between 1614 and 1429 cm^−1^ [39,40]; and C-O stretching at 1060 cm^−1^ [41]. Compared with glucose-derived HTCC photocatalysts reported in the previous literature [34], the straw-derived HTCC synthesized in this study showed a higher concentration of oxygen-containing functional groups on the surface, which can serve as the base sites in photocatalysis, and facilitate the formation of reductive active species such as the ·H radicals.

The Raman spectra of the pristine HTCC and 0.1 M Acid-HTCC are shown in Figure 1f. It is known that the D band (disordered band) centered at 1350 cm^−1^ usually represents the edges, defects, and disordered carbon sites, while the G band at located at 1580 cm^−1^ (graphite band) originates from the stretching motion of sp^2^-hybridized carbon atoms in the graphite structure [42]. Consequently, the concentration of surface defects for the as-prepared two samples can be investigated by comparing the intensity ratio of the D and G bands (I_D_/I_G_) in the spectra. Typically, a higher I_D_/I_G_ ratio indicates a higher concentration of surface defects. From the figure, both samples exhibited distinct D and G bands. The calculated I_D_/I_G_ values for the pristine HTCC and 0.1 M Acid-HTCC are 0.741 and 0.805, respectively, indicating that the acid modification introduced more defect sites, which could act as the trapping centers for photogenerated electrons in photocatalysis, thereby enhancing photocatalytic activity. Moreover, in contrast to 0.1 M Acid-HTCC, the spectrum of pristine HTCC showed a distinct shoulder peak at 1200 cm^−1^ [43,44]. This feature, assigned to sp^3^-hybridized carbon atoms of the cellulose, was eliminated by acid modification, confirming its role in promoting aromatization. Previous reports have confirmed that the semiconducting properties of HTCC photocatalysts are due to their sp^2^-hybridized structure of internal aromatic rings [29,34]. Therefore, the higher aromatization level caused by acid modification is beneficial to the enhancement of photocatalytic performances.

**Figure 2:**
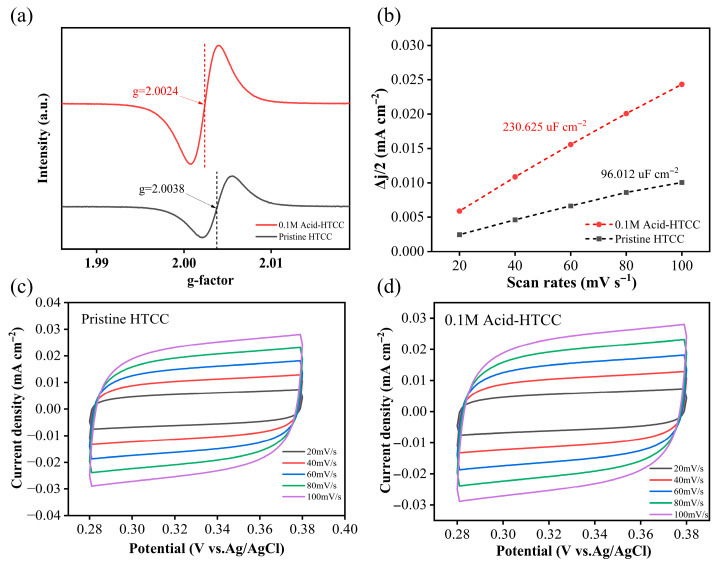
(**a**) ESR spectra and (**b**) C_dl_ values of the pristine HTCC and 0.1 M Acid-HTCC, respectively. CV curves of the (**c**) pristine HTCC and (**d**) 0.1 M Acid-HTCC at different scan rates.

It is known that carbon materials have been shown to harbor abundant persistent free radicals (PFRs) on their surfaces, enabling them to function as electron-trapping centers (i.e., active sites) in photocatalysis [45,46]. As shown in Figure 2a, distinct ESR signals of PFRs, presenting as singlets with respective g-factors of 2.0038 and 2.0024, were observed on both the pristine HTCC and 0.1 M Acid-HTCC photocatalysts. According to previous reports, singlet signals with g-values below 2.0030 are typically assigned to carbon-centered PFRs (C-PFRs), while those in the range of 2.0030–2.0040 can be attributed to carbon-centered persistent radicals in which the carbon atoms are adjacent to oxygen atoms (C-O PFRs). Thus, in this study, the pristine HTCC mainly contained C-O PFRs, whereas the 0.1 M Acid-HTCC was dominated by C-PFRs. The integration of the ESR spectra of 0.1 M Acid-HTCC revealed a higher concentration of PFRs compared with that of the pristine HTCC, which is consistent with the above Raman results, suggesting that the existence of its enhanced active sites that might facilitate the trapping of photogenerated electrons and promote photocatalytic activities.

According to above BET results, the specific surface area of the 0.1 M Acid-HTCC showed little difference from that of the pristine HTCC. To further investigate the concentration of the active sites involved in the photocatalytic reaction, the electrochemical active surface area (ECSA) was determined using the double-layer capacitance method [47] as follows:

ESCA = C_dl_/C_s_

where C_dl_ represents the double-layer capacitance derived from the linear fitting of capacitive currents in the non-Faradaic region of cyclic voltammetry (CV) curves at various scan rates; C_s_ is the specific capacitance of an ideal smooth surface under equivalent conditions. Figure 2c,d present the CV curves of HTCC and 0.1 M Acid-HTCC in the non-Faradaic region (0.28 V–0.38 V) at different scan rates. The charging current density (∆J) was obtained according to the average value of anodic current density and cathodic current density at the central potential in the scanning range. Then, the slopes of the fitted lines (scatter plot of ∆J versus scan rate) in Figure 2b were used as C_dl_ values for each catalyst, which were used to reflect the ECSA of the two samples. The C_dl_ value of 0.1 M Acid-HTCC was calculated to be 230.625 μF cm^−2^, which is 2.4 times greater than that of the pristine HTCC (90.012 μF cm^−2^). These results demonstrate that the acid-modified photocatalyst of 0.1 M Acid-HTCC possessed a larger electrochemical active surface area (i.e., more active sites) compared with the pristine HTCC, thereby contributing to its enhanced photocatalytic performances.

**Figure 3:**
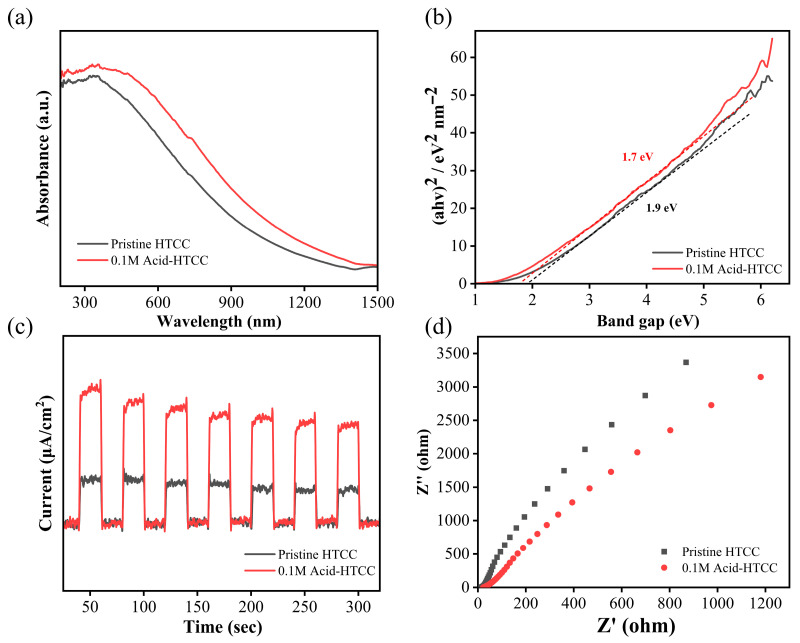
(**a**) UV–vis diffuse reflection spectra, (**b**) Kubelka–Munk curves, (**c**) photocurrent responses, (**d**) Nyquist plots of EIS for the pristine HTCC and 0.1 M Acid-HTCC.

The optical absorption properties and bandgap structures of the two photocatalysts were investigated using the UV–vis diffuse reflection spectra. As shown in Figure 3, the 0.1 M Acid-HTCC exhibited a significantly enhanced absorption capacity and a red-shifted absorption edge, which can be attributed to its higher degree of aromatization compared with the pristine HTCC. The bandgaps (E_g_) of two samples were determined using the Tauc plot method [48,49] as follows:

(αhυ) = A(hυ − E_g_)^n/2^ (1)

hυ = 1240/λ(2)

where α represents the absorption coefficient, h is the Planck constant, υ is the vibration frequency of light, A is a proportionality constant, and n is related to the semiconductor type [50]. The corresponding transformed Kubelka–Munk curves are presented in Figure 3b. The calculated E_g_ of 0.1 M Acid-HTCC was 1.70 eV, which was narrower than that of the pristine HTCC (1.90 eV), indicating that acid modification resulted in a reduced bandgap and enhanced light absorption, thereby enhancing the photocatalytic performances.

The separation efficiencies for the photogenerated carriers of the pristine HTCC and 0.1 M Acid-HTCC were investigated using the photocurrent and electrochemical impedance spectra (EIS) tests. As shown in Figure 3c, the photocurrent intensity of 0.1 M Acid-HTCC was approximately 2.5 times higher than that of the pristine HTCC, indicating the significantly improved separation efficiency of photogenerated carriers after acid modification. Moreover, the Nyquist plot in Figure 3d revealed a smaller semicircular arc radius for 0.1 M Acid-HTCC compared with the pristine HTCC, suggesting reduced electron transfer resistance and the more efficient separation of photogenerated carriers, which is consistent with the photocurrent measurements and ultimately enhanced its photocatalytic performance.

### 2.2. Photocatalytic Reduction of Cr(VI)

**Figure 4:**
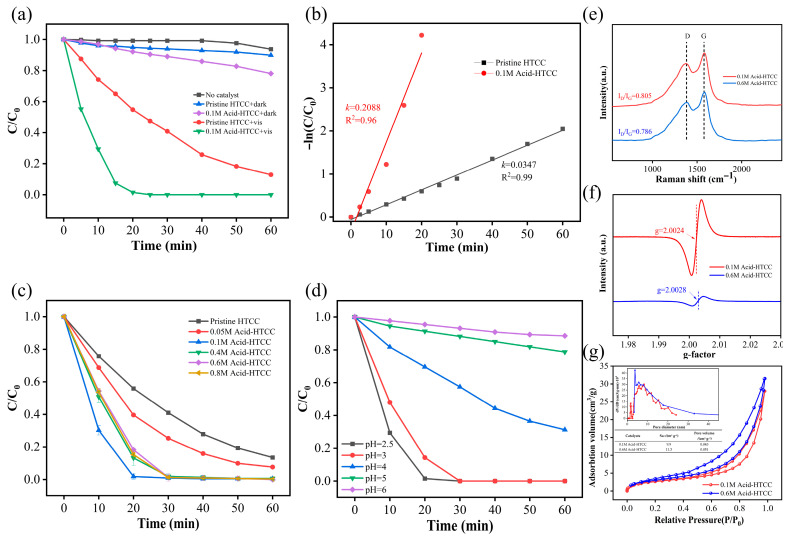
(**a**) Time profiles and (**b**) corresponding kinetic plots for the photocatalytic reduction of aqueous Cr(VI) (pH 2.5) with the pristine HTCC and 0.1 M Acid-HTCC under visible light irradiation. (**c**) Time profiles for the photocatalytic reduction of aqueous Cr(VI) (pH 2.5) with the HTCC photocatalysts treated with different acid concentrations under visible light irradiation. (**d**) Photocatalytic reduction of Cr(VI) under different pH conditions. Experimental conditions: catalyst concentration = 1.0 g L^−1^, pH = 2.5, Cr(VI) concentration = 20 mg L^−1^; (**e**–**g**) Raman, ESR, and N_2_ adsorption–desorption curves of the 0.1 M Acid-HTCC and 0.6M Acid-HTCC.

The photocatalytic performances of the prepared catalysts were first evaluated using aqueous Cr(VI) solution as the model pollutant. As shown in Figure 4a, the photolysis of Cr(VI) was negligible in the absence of a photocatalyst, and the adsorption removal of Cr(VI) was also very weak, removing only 10.1% and 21.9% of Cr(VI) for the pristine HTCC and 0.1 M Acid-HTCC after 60 min, respectively. The pristine HTCC displayed low photocatalytic activity for Cr(VI) reduction, yielding a Cr(VI) removal of 87.1% within 60 min irradiation. However, the Cr(VI) was completely removed within 20 min under identical experimental conditions when the 0.1 M Acid-HTCC was used as the photocatalyst. The photocatalytic reduction of Cr(VI) followed a first-order kinetic model, as presented in Figure 4b. The calculations demonstrated that the rate constant k for the photoreduction of Cr(VI) with the 0.1 M Acid-HTCC was 0.2088 min^−1^, being about 600% higher than that with pristine HTCC (0.0347 min^−1^). Therefore, the acid-modified photocatalyst of the 0.1 M Acid-HTCC exhibited a markedly enhanced photocatalytic performance. Moreover, the effect of acid concentration (0–0.8 mol L^−1^) on preparation on the photocatalytic performance was further investigated. Figure 4c shows the time profiles for the photocatalytic reduction of aqueous Cr(VI) (pH 2.5) with the HTCC photocatalysts treated with different acid concentrations under visible light irradiation. From the figure, the 0.1 M Acid-HTCC exhibited a superior performance, whereas higher acid concentrations (0.4–0.8 mol L^−1^) led to a decline in Cr(VI) removal efficiency.

To further investigate this phenomenon, comparative characterizations were conducted between 0.1 M Acid-HTCC and 0.6M Acid-HTCC (higher acid concentration sample) using the Raman, EPR, and N_2_ adsorption–desorption isotherms. From the Raman spectra in Figure 4e, the I_D_/I_G_ ratio of 0.6 M Acid-HTCC (0.786) is lower than that of the 0.1 M Acid-HTCC (0.805), indicating its reduced surface defect sites. Accordingly, as shown in Figure 4f, distinct ESR signals of C-PFRs with respective g-factors of 2.0024 and 2.0028 were observed with both the 0.1 M Acid-HTCC and 0.6 M Acid-HTCC photocatalysts. The ESR signal of 0.6 M Acid-HTCC exhibited a significant decrease compared with that of the 0.1 M Acid-HTCC, indicating its lower PFR concentration, which could be attributed to the cleavage of oxygen-containing functional groups adjacent to the PFR centers. Moreover, the values for the specific surface area and pore volume of 0.6 M Acid-HTCC (10.3 m^2^ g^−1^ and 0.045 cm^3^ g^−1^) did not differ significantly from that of the 0.1 M Acid-HTCC (9.9 m^2^ g^−1^ and 0.043 cm^3^ g^−1^), indicating that the addition of highly concentrated acid did not cause the evident collapse of the carbon structure in the catalyst (Figure 4g). Thus, the decline in photocatalytic performance at a high acid concentration can be mainly attributed to the lowered concentration of surface defects (i.e., PFRs), which serve as the critical active centers in reactions.

### 2.3. The Influence of pH

Since the reduction of Cr(VI) is generally considered a proton-coupled electron transfer (PCET) process, the influence of pH on Cr(VI) reduction with the acid-modified photocatalyst of 0.1 M Acid-HTCC was investigated. As shown in Figure 4d, the removal efficiency of Cr(VI) gradually decreased with increasing pH, as a higher proton concentration (lower pH) favors the reduction of Cr(VI). Therefore, the rate-determining step for Cr(VI) reduction by the HTCC photocatalysts in this work was governed by a PCET process rather than independent proton transfer (PT) or electron transfer (ET) processes.

### 2.4. Photocatalytic Degradation of Microcystin-LR

**Figure 5:**
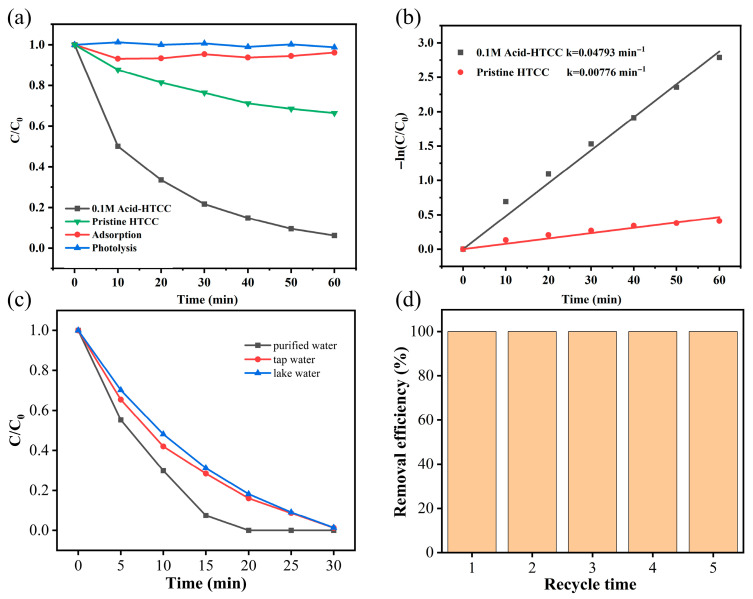
(**a**) Time profiles and (**b**) corresponding kinetic plots for the photocatalytic degradation of microcystin-LR with the pristine HTCC and 0.1 M Acid-HTCC under visible light irradiation. (**c**) Photocatalytic reduction of aqueous Cr(VI) with the 0.1 M Acid-HTCC in different water matrixes. (**d**) Cyclic experiments of the 0.1 M Acid-HTCC for Cr(VI) reduction.

Microcystin-LR is a highly toxic cyclic peptide produced and released by cyanobacteria during algal blooms in eutrophic waters [51,52,53]. The photocatalytic performances of the prepared catalysts were further investigated by the degradation of microcystin-LR aqueous solution under visible light irradiation. As shown in Figure 5a, similar to the Cr(VI) reduction above, the photolysis of microcystin-LR was negligible in the absence of a photocatalyst, and the adsorption removal (if any) of microcystin-LR was also very weak (only the adsorption on 0.1 M Acid-HTCC is shown in the figure). The pristine HTCC displayed low photocatalytic activity for microcystin-LR degradation, yielding a microcystin-LR removal of 33.6% within 50 min. However, the microcystin-LR removal within 50 min was increased to 93.9% when 0.1 M Acid-HTCC was used as the photocatalyst. The photocatalytic degradation of microcystin-LR followed a first-order kinetic model and the calculated rate constants are presented in Figure 5b. From the figure, the rate constant k for the degradation of microcystin-LR with the 0.1 M Acid-HTCC was 0.04793 min^−1^, which was about six-fold times higher than that with pristine HTCC (0.00776 min^−1^). These results are similar to the Cr(VI) reduction above. Additionally, the effect of catalyst dosage on the degradation of microcystin-LR was also discussed. As shown in Figure S3, the degradation efficiency of microcystin-LR with the 0.1 M Acid-HTCC increased with catalyst dosage from 0.4 to 1 g/L, and a further increase to 1.2 g/L did not improve the efficiency. The catalyst exhibited a superior photocatalytic performance at a catalyst dosage of 1 g/L. A higher dosage not only hampered light irradiation but also accelerated the recombination of photogenerated charge carriers.

### 2.5. The Influence of Water Matrix

The photocatalytic Cr(VI) reduction performance of the 0.1 M Acid-HTCC was evaluated in different water matrixes, including deionized water, tap water, and natural water from Nanhu Lake in Wuhan. As shown in Figure 5c, the removal efficiency of Cr(VI) was slightly decreased in tap water and natural lake water compared with that in deionized water due to the presence of coexisting oxidizing ions and impurities (such as nitrite and active chlorine), which quenched the photogenerated electrons and other active species. Notably, the complete removal of Cr(VI) was achieved within 30 min in all water matrices. This high effectiveness, even with a reduced removal rate, indicates that the acid-modified catalyst retains high activity in complex aqueous conditions and shows strong promise for practical use. Additionally, the photocatalytic performances of the 0.1 M Acid-HTCC for the removal of Cr(VI) and microcystin-LR were improved compared with other catalytic systems (Table S1) reported in previous studies [36,54,55,56,57,58].

### 2.6. Catalyst Stability

To assess its photocatalytic stability, the acid-modified catalyst (0.1 M Acid-HTCC) was subjected to five successive cycles of photocatalytic Cr(VI) reduction. Each cycle was conducted under identical reaction conditions, with a total catalyst mass loss of approximately 7% observed after the five cycles. As shown in Figure 5d, the 0.1 M Acid-HTCC retained an excellent reduction performance, achieving a near 100% photocatalytic reduction of Cr(VI) in each cycle. Moreover, no significant differences in the phase structure and morphology could be observed in the XRD patterns (Figure S4) and SEM images (Figures S1 and S5) of 0.1 M Acid-HTCC before and after the cyclic tests, demonstrating its high stability and reusability.

### 2.7. Photocatalytic Mechanism in Cr(VI) Reduction

**Figure 6:**
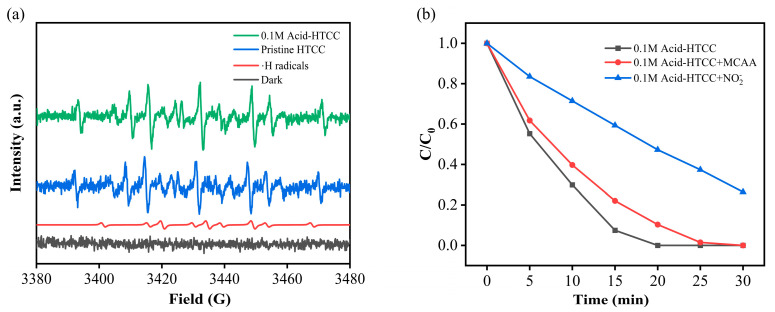
(**a**) DMPO spin-trapping ESR spectra of ·H radicals during the photocatalytic Cr(VI) reduction. (**b**) Photocatalytic reduction curves of Cr(VI) with the 0.1 M Acid-HTCC when NO_2_^−^ and MCAA were used as scavengers.

To investigate the roles of reactive species in photocatalytic Cr(VI) reduction, quenching experiments and ESR measurements with the DMPO as the trapping agent were conducted during Cr(VI) reduction with the pristine HTCC and 0.1 M Acid-HTCC. As shown in Figure 6a, under light irradiation, both pristine HTCC and 0.1 M Acid-HTCC yielded ESR signals with an intensity ratio of 1:1:2:1:2:1:2:1:1. Comparison with standard reference cards confirmed that the above detected signals originate from the ·H radicals [59]. No significant signals were detected under dark conditions. Moreover, the roles of key active species in the photocatalytic reduction of Cr(VI) with the 0.1 M Acid-HTCC were investigated using nitrite (NO_2_^−^) and chloroacetic acid (MCAA) as scavengers for ·H and hydrated electrons (e_aq_), respectively. As listed in Table 2 [60,61], NO_2_^−^ exhibited similar reaction rate constants with both e_aq_ and ·H, whereas MCAA reacted more readily with e_aq_ than with ·H. Thus, NO_2_^−^ can simultaneously quench both e_aq_ and ·H, while MCAA selectively scavenges e_aq_. As shown in Figure 6b, the addition of 5 mmol L^−1^ NO_2_^−^ suppressed the Cr(VI) reduction efficiency of 0.1 M Acid-HTCC from 100% to 73.63% within 30 min. In contrast, adding MCAA under identical conditions only resulted in a decrease of less than 8%. These results clearly indicated that the ·H played the dominant role in the photocatalytic reduction process of as-prepared HTCC photocatalysis.

## 3. Materials and Methods

### 3.1. Chemical Reagents

The straw used in this work was collected in Suqian City, Jiangsu province, China. Nitric acid (HNO_3_, 65–68%, analytical grade) was obtained from Xilong Science Co., Ltd. (Guangzhou, China). 5,5-Dimethyl-1-pyrrolidine-N-oxide (DMPO, 97%) was purchased from Aladdin Biochemical Technology Co., Ltd. (Shanghai, China). Anhydrous ethanol (C_2_H_6_O), potassium bromide (KBr), isopropanol ((CH_3_)_2_CHOH), Nafion (C_10_H_8_O), anhydrous sodium sulfate (Na_2_SO_4_), potassium dichromate (K_2_Cr_2_O_7)_, hydrochloric acid (HCl), barium sulfate (BaSO_4_), and 1,5-Diphenylcarbazide (C_13_H_14_N_4_O) were supplied by Sinopharm Chemical Reagent Co., Ltd. (Shanghai, China). All reagents were of analytical-grade purity. Deionized water was used throughout the experiments.

### 3.2. Synthesis of Catalysts

All the catalysts were synthesized through a hydrothermal method. Typically, the precursor of straw was first shredded into a fine powder using a mechanical grinder and then sieved to a specific particle size (100 mesh). Then, 3 g of the powder was added into 60 mL of nitric acid solution with different concentrations. After intensively stirring for 30 min, the above suspension was transferred into a stainless-steel Teflon-lined autoclave of 100 mL capacity. The autoclave was sealed and heated at 180 °C for 12 h and then cooled to room temperature. The acquired precipitate was collected by centrifugation, washed with deionized water and ethanol for 5 times, and then dried in a vacuum oven at 60 °C for 12 h. The obtained samples were named as xM Acid-HTCC, where x represents the concentration of nitric acid solution (0.05, 0.1, 0.4, or 0.8 mol L^−1^). For comparison, pristine HTCC was prepared under identical conditions without the addition of nitric acid.

### 3.3. Characterization

The structure and phase of the as-prepared samples were characterized by X-ray diffraction (XRD; D8 ADVANCE, Cu Kα radiation) from Bruker Daltonics GmbH & Co., located in Bremen, Germany. The Brunauer–Emmett–Teller (BET) data were measured by N_2_ adsorption–desorption isotherms obtained at 77 K on an Autosorb-iQ (Quantachrome, Boynton Beach, FL, USA) after an overnight vacuum degassing process. Scanning electron microscopy (SEM) images were acquired on a S-4800 system operated at a 1.5 kV decelerating voltage (Hitachi, Tokyo, Japan). Chemical bonds of samples were analyzed using Fourier transform infrared (FTIR, INVENIO-R) spectroscopy with an MCT detector (Bruker Daltonics, Bremen, Germany). Raman spectra of samples were collected using a DXR Raman microscopy system with an excitation wavelength of 532 nm (Thermo Fisher Scientific, Waltham, MA, USA). ESR measurements were performed using a ESP 300 E spectrometer with a sweep width of 1000 Gs (Bruker Daltonics, Bremen, Germany). UV–vis diffuse reflectance spectra (DRS) were detected by a UV2600 spectrophotometer, using BaSO_4_ as the reflectance standard (Shimadzu, Kyoto, Japan). Photocurrent and electrochemical impedance spectroscopy (EIS) in Na_2_SO_4_ (0.1 mol L^−1^) aqueous solution were analyzed using a CHI660 electrochemical work-station (CH Instruments, Shanghai, China) with a standard three-electrode cell, where 20 mg of the as-prepared catalyst sample powders was sonicated in a mixture of 0.5 mL isopropanol and 20 μL Nafion solution and anhydrous ethanol for 4 h. Then, 20 μL of the suspensions was dripped onto a 1 × 1 cm^2^ size FTO conductive glass as the working electrode, a Pt electrode as the auxiliary electrode, and a standard Ag/AgCl as the reference. A 350 W xenon lamp was served as the light source.

### 3.4. Photocatalytic Experiments

The photocatalytic reduction of aqueous Cr(VI) was conducted under visible light irradiation at room temperature (25 °C). Briefly, aqueous Cr(VI) solution (20 mg L^−1^, pH 2.5) was prepared by diluting the K_2_Cr_2_O_7_ stock solution (100 mg L^−1^) with deionized water. Then, after adding the catalyst (20 mg) into 20 mL of the above Cr(VI) solution, the suspension was sonicated (40 kHz, 5 min) to disperse the catalyst uniformly and magnetically stirred (30 min) in the dark to achieve adsorption–desorption equilibrium. Irradiation was then initiated using a 300 W Xe lamp with a 420 nm cutoff filter. At given time intervals, 1 mL aliquots were withdrawn and analyzed for Cr(VI) concentration via the standard diphenylcarbazide method.

For the degradation of microcystin-LR, the experiments were performed as follows: 20 mg of the as-prepared samples was added in 20 mL of microcystin-LR solution with the initial concentration of 0.5 mg L^−1^. Prior to irradiation, the suspension was stirred for 30 min in the dark to reach adsorption–desorption equilibrium. The reaction temperature was kept at room temperature to prevent any thermal catalytic effects. At given time intervals, 0.5 mL of suspension was placed in vials and analyzed with high-performance liquid chromatography (HPLC, 1260 infinity) for the quantification of microcystin-LR (Agilent, Santa Clara, CA, USA). For the HPLC analysis, the injection volume to a Diamonsil C-18 column was 20 μL. The mobile phase in isocratic mode with a flow rate of 0.2 mL min^−1^ was a mixture of trifluoroacetic acid aqueous solution and methanol at a volume ratio of 13:7. The microcystin-LR was measured with a photodiode array detector at 238 nm.

## 4. Conclusions

In summary, in this work, acid-modified HTCC photocatalysts were prepared via a one-step hydrothermal process with straw as the precursor. On the basis of various characterizations, we confirmed that the acid-modified HTCC photocatalyst exhibited a higher aromatization level, enhanced light-harvesting ability, and increased concentration of active sites compared with the pristine HTCC. The photocurrent and electrochemical impedance spectra (EIS) experiments indicated that the acid modification of HTCC remarkably promoted the separation efficiency of photogenerated carriers. As a result, the photocatalytic removal rate of Cr(VI) and microcystin-LR with the acid-modified HTCC photocatalyst were dramatically enhanced, both being about six-fold times higher than that with pristine HTCC. Moreover, the as-prepared acid-modified HTCC photocatalysts exhibited a high removal efficiency of Cr(VI) in different water matrixes. Only a slight decrease in efficiency was observed within 30 min in both tap water and natural lake water. Based on the ESR and quenching experiments, ·H radicals were identified as the dominant active species in the photocatalytic reduction process. Additionally, the acid-modified HTCC demonstrated excellent reusability and stability, with only a negligible loss in degradation efficiency and minimal change in structural integrity after five consecutive cycles. Consequently, our findings provide valuable insights for the application of biomass-derived photocatalysts in resource circulatory utilization and environmental remediation.

## Figures and Tables

**Table 1:** Specific surface area, total pore volume, and average diameter of the pristine HTCC and 0.1 M Acid-HTCC.

Catalysts	S_BET_/(m^2^ g^−1^)	Pore Volume/(cm^3^ g^−1^)	Pore Size/(nm)
Pristine HTCC	6.6	0.032	17.3
0.1 M Acid-HTCC	9.9	0.043	8.3

**Table 2:** Rate constants of e_aq_ and ·H radical scavengers.

Scavenger	Rate Constants (mol^−1^ s^−1^)	
	e_aq_^−^	·H
NO_2_^-^	3.5 × 10^9^	7.1 × 10^8^
MCAA	6.9 × 10^9^	6.5 × 10^3^

## Data Availability

The original contributions presented in this study are included in the article/Supplementary Material. Further inquiries can be directed to the corresponding author(s).

## Supplementary Materials

The following supporting information can be downloaded at: https://www.mdpi.com/article/10.3390/molecules30224399/s1, Figure S1. SEM images of the (a) pristine HTCC and (b) 0.1 M Acid-HTCC. Figure S2. (a) TEM and (b) HRTEM images of the 0.1 M Acid-HTCC. Figure S3. Time profiles for the photocatalytic degradation of microcystin-LR with the 0.1 M Acid-HTCC with different catalyst dosages under visible light irradiation. Figure S4. XRD patterns of 0.1 M Acid-HTCC before and after the cyclic tests. Figure S5. SEM images of 0.1 M Acid-HTCC after the cyclic tests. Table S1. Comparison of the photocatalytic performances with the literature. Refs. [36,54,55,56,57,58] are cited in the Supplementary Materials.
