# Supplementary material for: Synthesis of Straw-Based Hydrothermal Carbonation Carbon and Its Photocatalytic Removal of Cr(VI) and Microcystin-LR
Source: Molecules. 2025 Nov 14;30(22):4399. doi: 10.3390/molecules30224399 (PMC12655307; doi:10.3390/molecules30224399)
Supplement: Supplementary file 1 [file molecules-30-04399-s001.zip › molecules-3965519-supplementary.docx]

**Electronic Supplementary Information**

**Synthesis of straw-based hydrothermal carbonation carbon and its photocatalytic removal of Cr(VI) and microcystin-LR**

Yu Luo ^1^, Xunxian Chen ^2^, Zhen Wan ^1^*and Yingming Chen ^1^*

^1^ Key Laboratory of Catalysis and Materials Science of the State Ethnic Affairs Commission and Ministry of Education, College of Resources and Environmental Science, South-Central Minzu University, Wuhan 430074, China;

^2^ Foshan Water Industry Group Co., Ltd. Foshan 528000, China ;

**^*^** Correspondence: [2018030@scuec.edu.cn](mailto:2018030@scuec.edu.cn); Tel.: +86-27-67843990


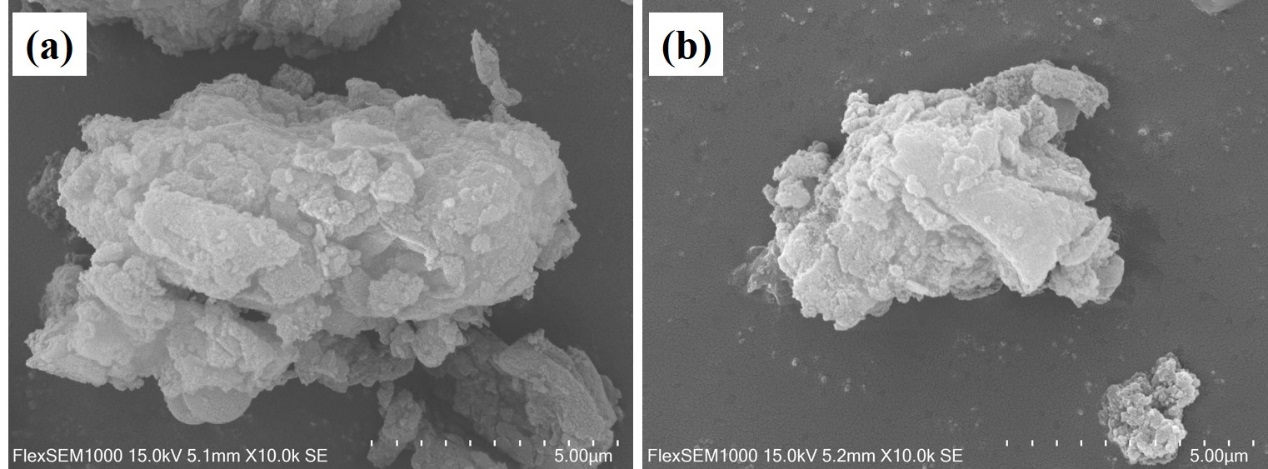


**Figure S1.** SEM images of the (a) pristine HTCC and (b) 0.1M Acid-HTCC respectively.


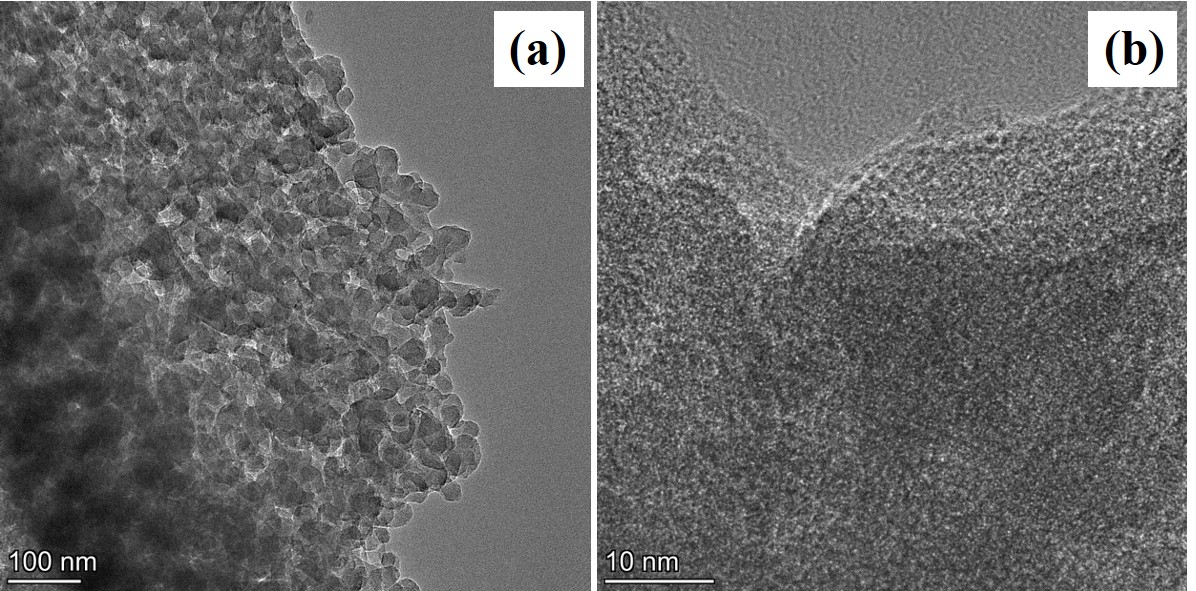


**Figure S2.** (a) TEM and (b) HRTEM images of the 0.1M Acid-HTCC.


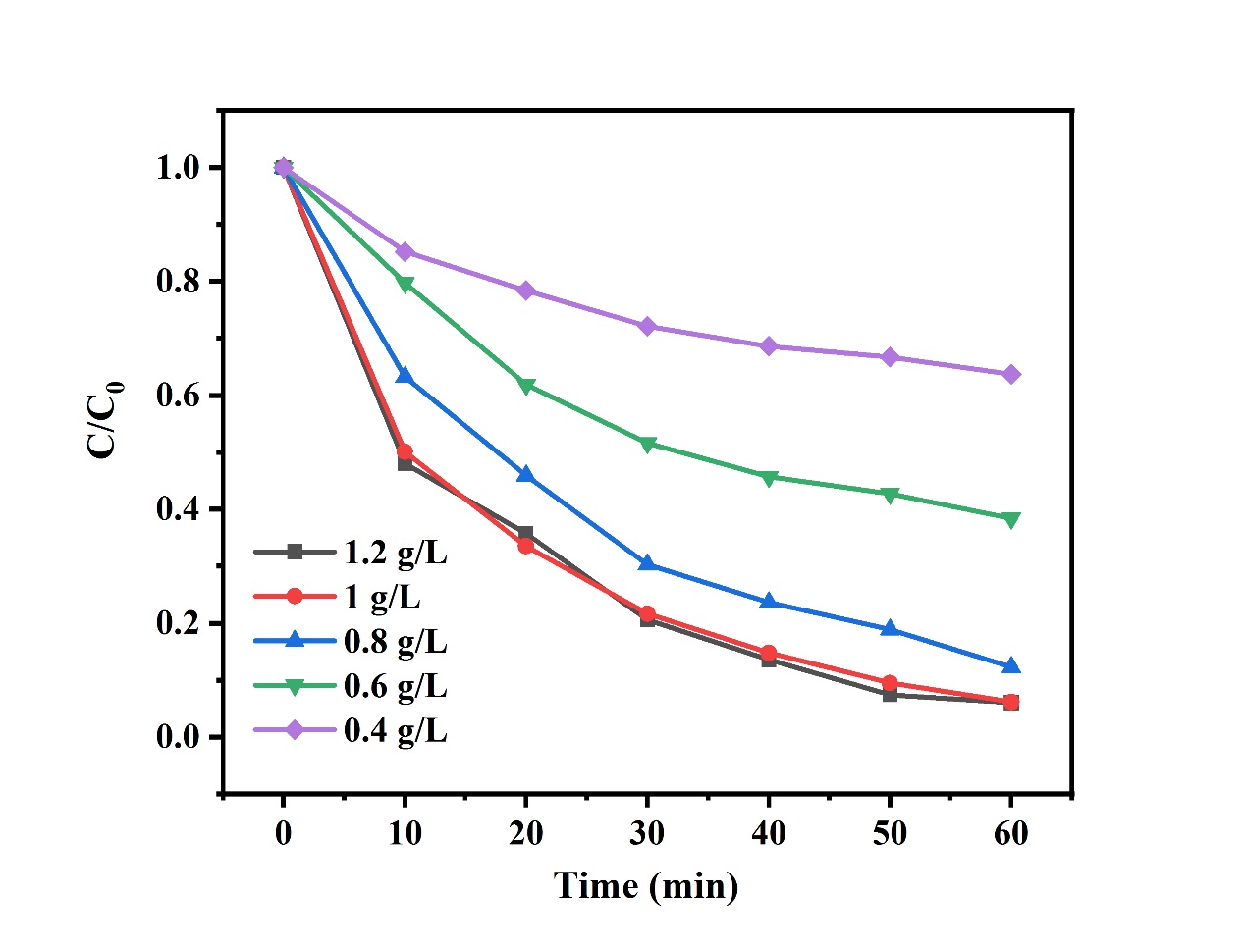


**Figure S3.** Time profiles for the photocatalytic degradation of microcystin-LR over the 0.1M Acid-HTCC with different catalyst dosages under visible light irradiation.


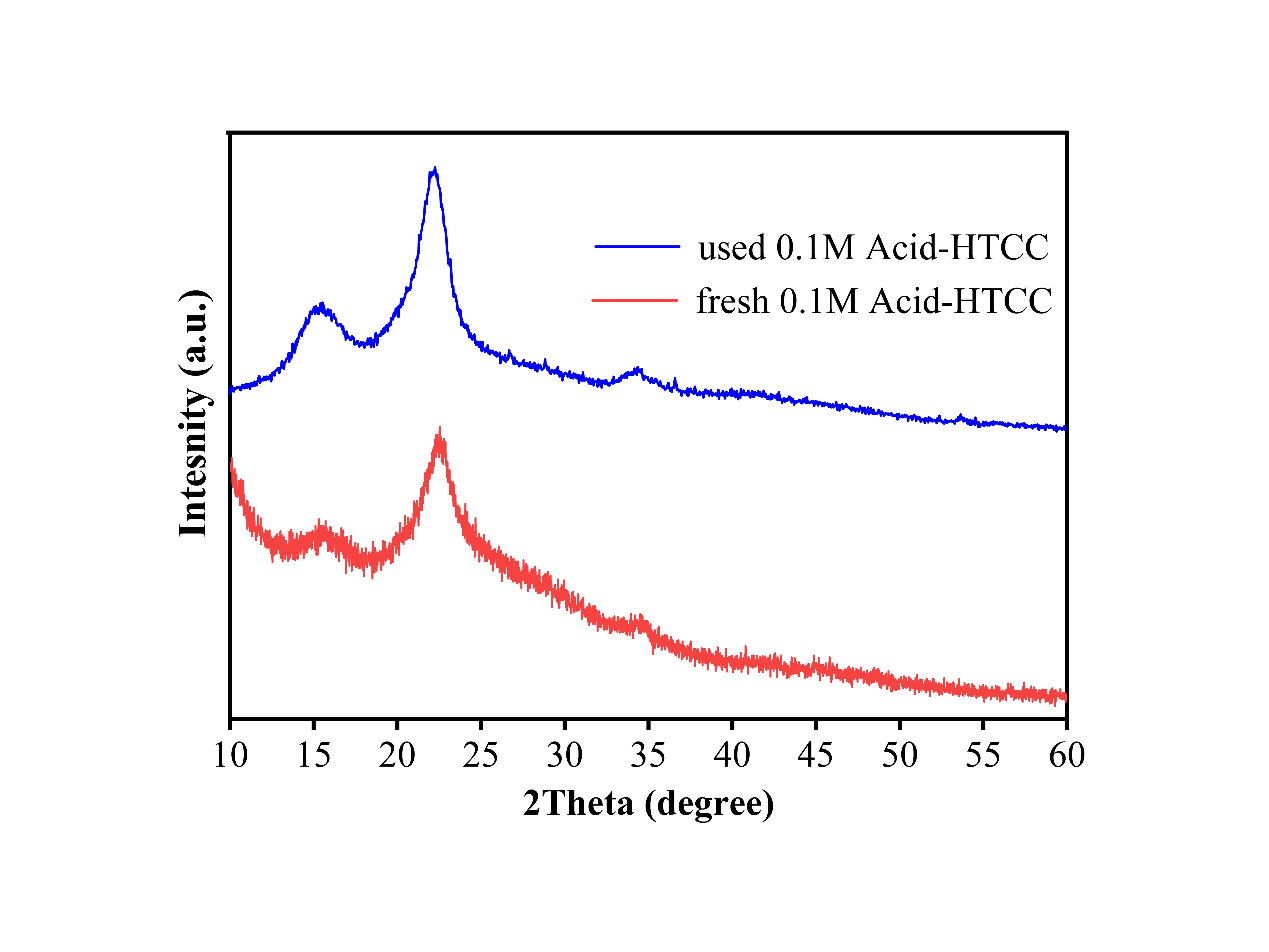


**Figure S4.** XRD patterns of 0.1M Acid-HTCC before and after the cyclic tests.


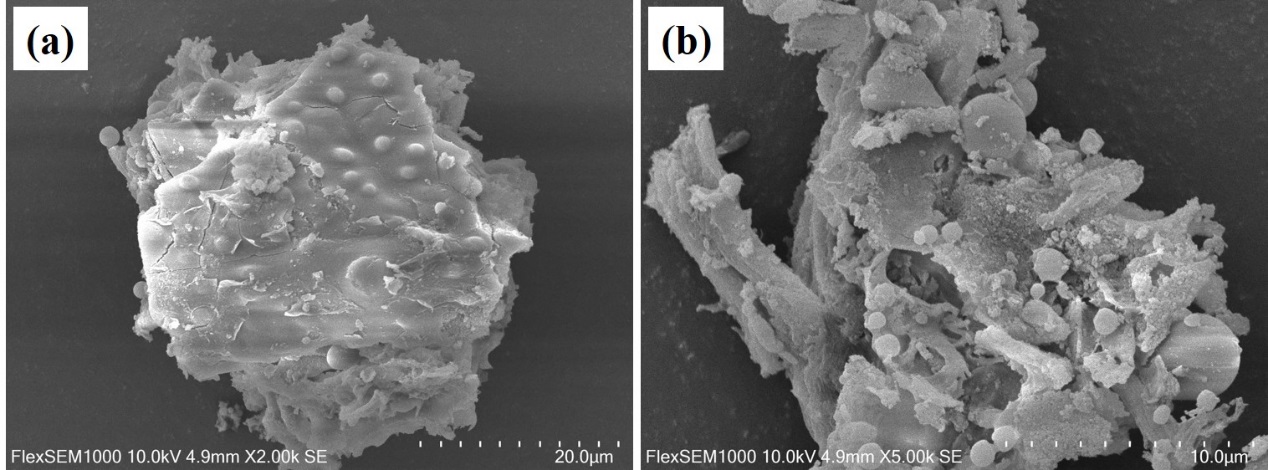


**Figure S5.** SEM images of 0.1M Acid-HTCC after the cyclic tests.

**Table S1.** The comparison of the photocatalytic performances with other literature.

| **Catalyst** | **Pollutants** | **Reaction conditions** | **Removal efficiency (%)** | **Ref.** |
| --- | --- | --- | --- | --- |
| 0.1M Acid-HTCC | Cr(VI) | [Catalyst] = 0.2 g L^-1^;  [Cr(VI)] = 20 mg L^-1^ | 100% (50 min) | This work |
| Acid-HTCC (Paperboard) | Cr(VI) | [Catalyst] = 0.142 g L^-1^;  [Cr(VI)] = 23.5 mg L^-1^ | 58 % (120 min) | [36] |
| HTC/g-C_3_N_4_ | Cr(VI) | [Catalyst] = 0.2 g L^-1^;  [Cr(VI)] = 20 mg L^-1^ | 70 % (100 min) | [54] |
| g-C_3_N_4_/ZFO | Cr(VI) | [Catalyst] = 0.5 g L^-1^;  [Cr(VI)] = 20 mg L^-1^ | 54 % (200 min) | [55] |
| g-C_3_N_4_/ZnIn_2_S_4_ | Cr(VI) | [Catalyst] = 0.1 g L^-1^;  [Cr(VI)] = 25 mg L^-1^ | 60 % (60 min) | [56] |
| 0.1M Acid-HTCC | Microcystin-LR | [Catalyst] = 1 g L^-1^;  [Microcystin-LR] = 0.5 mg L^-1^ | 93.9 % (50 min) | This work |
| Ag/BiOI | Microcystin-LR | [Catalyst] = 5 g L^-1^;  [Microcystin-LR] = 2.5 g L^-1^ | 61.26 % (120 min) | [57] |
| C-TiO_2_ | Microcystin-LR | [Catalyst] = 0.2 g L^-1^;  [Microcystin-LR] = 10 mg L^-1^ | 55 % (300 min) | [58] |

References

36. Xu, L.; Liu, Y.; Hu, Z.; Yu, J. C. Converting cellulose waste into a high-efficiency photocatalyst for Cr(VI) reduction via molecular oxygen activation. *Appl. Catal. B-Environ. Energy*. **2021**, 295, 120253. <https://doi.org/10.1016/j.apcatb.2021.120253>

54. Ding, X.; Xiao, D.; Ji, L.; Jin, D.; Dai, K.; Yang, Z.; Wang, S.; Chen, H. Simple fabrication of Fe_3_O_4_/C/g-C_3_N_4_ two-dimensional composite by hydrothermal carbonization approach with enhanced photocatalytic performance under visible light. Catal. Sci. Technol. **2018**, *14*, 3484-3492. <https://pubs.rsc.org/en/content/articlelanding/2018/cy/c8cy00698a>.

55. Wang, X.; Dai, Y.; Tian, C.; Zhang, H.; Li, X.; Liu, W.; Li, W.; Kuang, S.; Tong, H. Boosted photocatalytic removal of Cr(VI) using MoS_2_ modified g-C_3_N_4_/ZnFe_2_O_4_ magnetic heterojunction composites. Process Saf. Environ. Protect. **2022**, 162, 72-82. <https://doi.org/10.1016/j.psep.2022.04.005>.

56. Hu, J.; Lu, S.; Ma, J.; Zhu, F.; Komarneni, S. Composite of g-C_3_N_4_/ZnIn_2_S_4_ for efficient adsorption and visible light photocatalytic reduction of Cr(VI). Environ Sci Pollut Res. **2022**, 29, 76404-76416. https://doi.org/10.1007/s11356-022-21224-8.

57. Han, C.; Pelaez, M.; Likodimos, V.; Kontos, A. G.; Falaras, P.; O’Shea. K.; Dionysiou, D. D. Innovative visible light-activated sulfur doped TiO_2_ films for water treatment. *Appl. Catal. B-Environ.* **2011**, 107, 77-87. <https://doi.org/10.1016/j.apcatb.2011.06.039>.

58. Zhang, F.; Peng, H.; Jiang, S.; Wang, C.; Xu, X.; Wang, L. Construction of precious metal-loaded BiOI semiconductor materials with improved photocatalytic activity for microcystin-LR degradation. *Environ. Sci. Pollut. Res.* **2019**, 26, 8226-8236. https://doi.org/10.1007/s11356-019-04266-3.
